# Preparation of Ecofriendly Formulations Containing Biologically Active Monoterpenes with Their Fumigant and Residual Toxicities against Adults of* Culex pipiens*

**DOI:** 10.1155/2016/8540830

**Published:** 2016-11-07

**Authors:** Mohamed E. I. Badawy, Nehad E. M. Taktak, Osama M. Awad, Souraya A. Elfiki, Nadia E. Abou El-Ela

**Affiliations:** ^1^Department of Pesticide Chemistry and Technology, Faculty of Agriculture, Alexandria University, El-Shatby, Alexandria 21545, Egypt; ^2^Department of Tropical Health, High Institute of Public Health, Alexandria University, Alexandria, Egypt

## Abstract

Different mixtures of monoterpenes (ketone, alcohol, and alkene) were loaded on paper discs and wax and their knockdown activities were evaluated against* Culex pipiens *adults. Some individual monoterpenes were also evaluated by residual toxicity technique. Citronella oil as a reference was also loaded separately or in combination with monoterpenes on paper discs and wax. The ketone monoterpenes mixture (camphor, menthone, carvone, and fenchone) on paper discs was the most active (KT_50_ = 17.20 min) followed by ketone monoterpenes with citronella oil (KT_50_ = 20.79 min) and citronella oil alone (KT_50_ = 28.72 min). Wax formulations proved that the ketone and alcohol (geraniol, thymol, and menthol) monoterpenes gave the most activity as knockdown (KT_50_ = 31.79 and 43.39 min, resp.). Alcohol monoterpenes formulation recorded KT_50_ = 43.39 min. Residual activity of tested individual monoterpenes reported that the menthol was more toxic than camphor and camphene. Generally, this study suggests that the monoterpenes have the properties, which make them used as eco-friendly compounds in the control programs of* Cx. pipiens *adult. The use of paper discs is more applicable than wax in the adulticidal formulations.

## 1. Introduction

Man has suffered from the activities of mosquitoes since time immemorial and it is ranked as man's most important insect pest. The genera of mosquito have been incriminated as the main vectors:* Culex, Aedes*, and* Anopheles*, which transmit several infectious diseases to human; for example, filariasis, Japanese encephalitis, dengue and yellow fever viruses, and malaria [1, 2] are major threat to over two billion people in the tropics. Mosquito bites may also cause allergic responses including local skin reactions and systemic reactions such as urticaria and angioedema [3].

In Egypt, the widespread house mosquito* Cx. pipiens molestus* (Forsk) has been recorded in all governorates without any exception [4] causing a health problem and nuisance to humans [5]. It is the major vector of bancroftian filariasis, which caused the problems of about 120 million infected and disabled persons annually [6], Rift valley fever, and diseases caused by other viruses [7, 8].

Mosquito control represents an important strategy for prevention of diseases transmission and epidemic outbreaks. Synthetic pesticides have been commonly used for adult mosquito control such as organophosphates (Malathion and Naled) and synthetic pyrethroids (Permethrin, Resmethrin, Sumithrin, Prallethrin, and Etofenprox). Adulticides were applied as ultra-low volume (ULV) sprays either by aircraft or on the ground employing truck-mounted sprayers. ULV sprayers dispense very fine aerosol droplets that stay aloft and kill flying mosquitoes on contact. ULV applications involve small quantities of pesticide active ingredient in relation to the size of the area treated, which minimizes exposure and risks to people and the environment [9, 10]. However, continued use of adulticides for generations develops the phenomenon of resistance accompanied by harmful effects on human health and the environment [1, 11–13]. The search for ecofriendly alternative mosquitocides that have minimal risk to human health and the environment is considered the goal of many researchers. Therefore, botanical insecticides have become more popular as alternatives to synthetic chemical mosquitocides, substantiated by many studies confirming the mosquitocidal properties of numerous plant-derived compounds from various sources [14–18]. Monoterpenes are the main component in the plurality of plant essential oils and give plants their unique odoriferous properties because of their low boiling points. The natural pesticidal properties of some monoterpenes make them good alternative pest control agents as well as good lead compounds for the development of ecofriendly and fully biodegradable pesticides. Some studies have been conducted on the insecticidal properties of monoterpenes against various mosquitoes. For example, Radwan and others studied fourteen major monoterpenoids and reported their toxic effect against* Cx. pipiens* [19]. The larvicidal and antioviposition effects of pulegone, thymol, and eugenol against* Ae. aegypti* were also described [20]. Zahran and Abdelgaleil evaluated twelve monoterpenes for larvicidal and adulticidal activities towards* Cx. pipiens* [5]. Knockdown and larvicidal activity of six monoterpenes were studied against* Ae. aegypti* and their structure-activity relationships were investigated [21]. Michaelakis et al. tested twenty acyclic monoterpenes with different functional groups as repellent and larvicidal agents against* Cx. pipiens* [22].

Therefore, the main goal of this study was to determine the efficiency of mixtures of some monoterpenes that have already toxic potential effect against mosquitoes. The study was conducted by two methods including residual and fumigant activity assays. The compounds were formulated in two types including impregnating them into paper discs and wax. The toxicity assessment was investigated as knockdown with calculation of the KT_50_ values and was discussed in detail.

## 2. Materials and Methods

### 2.1. Chemicals, Monoterpenes, and Essential Oil

**Figure 1:**
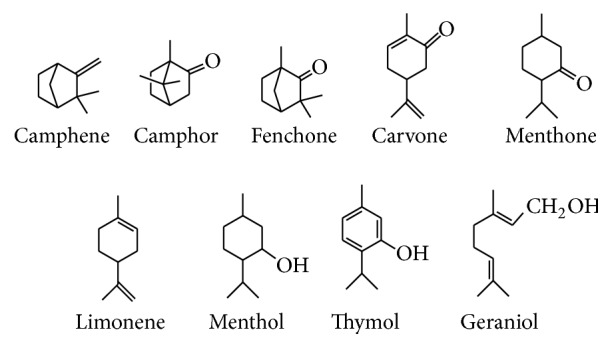
Chemical structures of pure tested monoterpenes.

**Figure 2:**
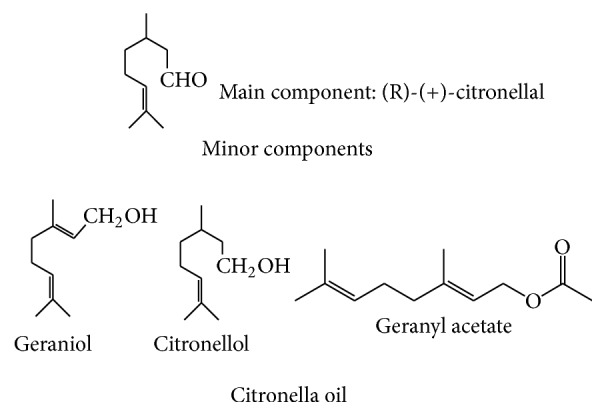
Constituents of citronella oil (R-(+)-citronellal as a main component and geraniol, citronellol, and geranyl acetate as minor ones).

Monoterpenes include (*R*)-camphor (98%), (*L*)-menthone (97%), (*S*)-fenchone (98%), (*R*)-carvone (98%), geraniol (98%), thymol (98%), (*1R, 2S, 5R*)-menthol (98%), (*S*)-limonene (96%), and camphene (95%) which were purchased from Sigma-Aldrich (St. Louis, MO, USA). Citronella oil was supplied from El Gomhoria Co. (Alexandria, Egypt). Chemical structures of these monoterpenes and citronella oil are shown in Figures 1 and 2, respectively. All other commercially available solvents and reagents were used without further purification.

### 2.2. Test Insect and Rearing

Third instar larvae of* Cx. pipiens* were obtained from Research Institute of Medical Entomology, Ministry of Health, Dokki, Giza, Egypt, and reared under laboratory conditions. About 400–600 larvae were transferred to white enameled and shallow trays about 30 cm in diameter containing 2-3 L of dechlorinated water. These trays were always covered with mesh screen to prevent oviposition by escaping adult mosquitoes and were maintained at a room temperature (26 ± 2°C) and RH (70 ± 5%) with a 14 : 10 (L : D) photo-period and water was replaced every two days. Larvae were daily fed on biscuits and yeast powder (3 : 1 ratio) until pupation. The pupae were transferred from the trays to plastic cups containing dechlorinated water into cages with netting cover wood frames (30 × 30 × 30 cm) until adults emerged. Adults were provided with 30% sucrose solution and females were fed on pigeon blood for four times a week [23]. The egg-rafts were placed in the white trays containing dechlorinated water for larval hatch.

### 2.3. Preparation of Ecofriendly Formulations

#### 2.3.1. Formulation of Biologically Active Monoterpenes Impregnated in Paper Discs

**Figure 3:**
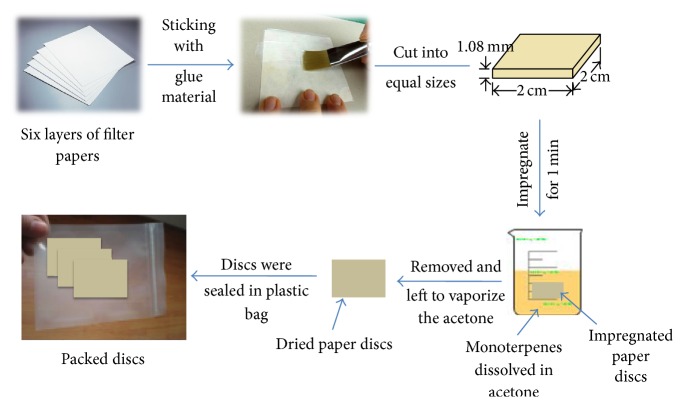
Schematic illustration of the preparation paper mosquito repellent incense.

The method used was according to Kawada and his coauthors [24, 25] with some modifications as follows: multilayer paper discs were prepared by sticking of six layers of filter paper (Whatman filter paper number 1) by glue material to form discs (2 × 2 cm, 1.08 mm average in thickness), and total surface area of the paper discs was 4 cm^2^. A weight of 100 mg of each monoterpene was dissolved in 10 mL acetone to obtain 10000 mg/L of each compound in final mixture solution. The paper discs (15 discs per replicate per each mixture) were dipped for one minute in this blend solution, then removed, and left to vaporize the acetone under ambient conditions. The discs were sealed in plastic bag (Figure 3). Seven formulations were prepared manually with average of 15 replicates for each formulation and then we calculated the loaded monoterpenes of each formulation according to the weight before each average of the discs. The formulated paper discs were used in bioassay to calculate the times required to knock down 50% of the female adults exposed (KT_50_). According to the class of monoterpenes, the paper discs divided into seven groups as PD1–PD7 (PD: paper discs) as follows:

immobilization of blend of ketone monoterpenes (camphor, menthone, carvone, and fenchone) on paper discs.
immobilization of blend of alcohol monoterpenes (geraniol, thymol, and menthol) on paper discs.
immobilization of blend of alkene monoterpenes (limonene and camphene) on paper discs.
PD1 + citronella oil on paper discs.
PD2 + citronella oil on paper discs.
PD3 + citronella oil on paper discs.
immobilization of citronella oil on paper discs.

#### 2.3.2. Formulation of Biologically Active Monoterpenes Incorporated on Wax

**Figure 4:**
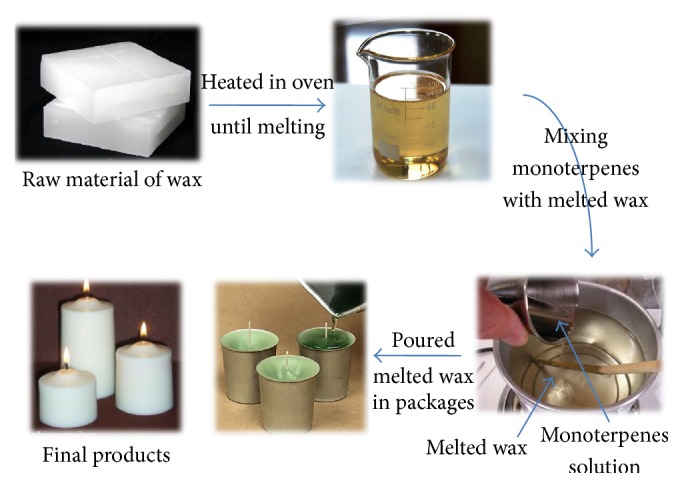
Schematic illustration of the preparation of monoterpenes incorporated into wax for mosquito control.

The sustained release of biologically active monoterpenes from wax was evaluated. The method of preparation was as follows: raw wax (50 g) was weighted and melted at 120°C. Blend of different tested monoterpenes dissolved in acetone was mixed with the melted wax to obtain 1000 ppm of each compound in final formulation. The solution was then poured into alumina cups with fuse (Figure 4). According to the class of monoterpenes, these products divided into seven groups W1–W7 mentioned previously (W: wax):

immobilization of blend of ketone monoterpenes (camphor, menthone, carvone, and fenchone) on paper discs.
immobilization of blend of alcohol monoterpenes (geraniol, thymol, and menthol) on paper discs.
immobilization of blend of alkene monoterpenes (limonene and camphene) on paper discs.
PD1 + citronella oil on paper discs.
PD2 + citronella oil on paper discs.
PD3 + citronella oil on paper discs.
immobilization of citronella oil on paper discs.

After solidification, the formulated products were used in bioassay to calculate KT_50_ against adult mosquitoes.

### 2.4. Adulticidal Bioassay Techniques

Susceptibility of* Cx. pipiens* female adult to different monoterpenes was assayed in two different formulations

#### 2.4.1. Fumigant Assay of Monoterpenes Loaded on Paper Discs against Female Adults

**Figure 5:**
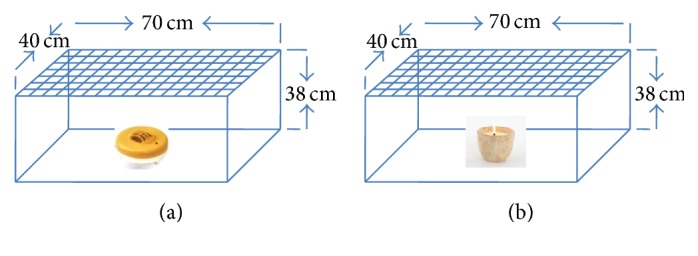
Fumigant assay of* paper discs against mosquito adults* (a)* and* knockdown assay of monoterpenes incorporated on wax (b).

The vapor action released from paper discs impregnated with biologically active monoterpenes was observed against adults of* Cx. pipiens* (Figure 5(a)). The paper disc was held on heater in the centre of the test chamber (70 × 40 × 38 cm). The paper discs were adapted to heat in the presence of 30 susceptible female adults. The number of knocked-down adults was counted at 5 min intervals along 60 minutes [25, 26]. Experiments were run in three replicates and the KT_50_ values were calculated according to the probit analysis [27].

#### 2.4.2. Fumigant Bioassay of Monoterpenes Incorporated on Wax

Evaluation of the vapor action of monoterpenes loaded into wax was tested against female adults of* Cx. pipiens* as shown in Figure 5(b). In test chamber (70 × 40 × 38 cm), the monoterpene candle was placed in the centre of the chamber and illuminated it in the presence of 30 female adults. The number of knocked-down mosquitoes was counted at 5 min intervals for 60 minutes. Experiments were run in three replicates and the KT_50_ values were calculated according to the probit analysis [27].

#### 2.4.3. Residual Activity of Monoterpenes against Female Adult of* Cx. pipiens*

The monoterpenes (camphor, menthol, and camphene) were selected from each tested group previously. Four concentrations (5, 10, 20, and 40 mg/cm^2^) of monoterpenes were prepared in acetone. Glass bottles 30 mL were coated with selected monoterpenes to form residual layer according to the Centre for Disease Control and Prevention (CDC) protocol [28]. The monoterpene left a sufficient period for the completion of drought. Twenty female adults were introduced into three glass bottles of 30 mL each, coated with monoterpenes. One control bottle of 30 mL was coated with acetone only. Deltamethrin at different concentrations (0.05, 0.1, 0.5, and 5 mg/cm^2^) was tested as a reference. The number of knocked-down mosquitoes was counted at 5 min intervals for 60 minutes. Experiments were run in three replicates. KT_50_ values were calculated by the probit method [27].

## 3. Results and Discussion

### 3.1. Formulation of Biologically Active Monoterpenes Impregnated in Paper Discs

In this study we prepared seven formulations of paper discs impregnated in different mixtures of monoterpenes and citronella oil. We divided the compounds according to their chemical groups to ketone, alcohol, and alkene monoterpenes groups avoiding any chemical reaction that may occur between them after loading into formulation. In addition, citronella oil was used as a reference because it is currently on the U.S. Environmental Protection Agency (EPA) list of minimum risk pesticides. Besides, it is considered one of the most essential oils extensively used in the markets as a repellent of mosquitoes. The properties of these paper discs are shown in Table 1. The concentration of the active ingredient (8.86%) in PD4 (camphor, menthone, carvone, fenchone, and citronella oil) formulation was the highest followed by PD5 (geraniol, thymol, menthol, and citronella oil) (8.51%). On the contrary, the concentration of the active ingredient in PD7 (citronella oil alone) formulation was the least (5.4%).

### 3.2. Fumigant Assay of Monoterpenes Loaded on Paper Discs against Female Adults

Knockdown activity of a mixture of monoterpenes impregnated paper discs against the female adult of* Cx. pipiens* is shown in Table 2. The results showed that after 1 hr exposure period at paper discs impregnated with different monoterpenes, the mixture of ketone monoterpenes (PD1) recorded the highest median time (KT_50_) which was calculated as 17.2 min followed by mixture of ketone monoterpenes and citronella oil (PD4) which was calculated as 20.8 min. The mixture of alkene monoterpenes (PD3) recorded knockdown median time at 27.4 min, followed by the paper disc impregnated with citronella oil alone (PD7) at 28.7 min and the mixture of alkene monoterpenes with citronella oil together (PD6) at 33.2 min. The lowest median time recorded with paper discs impregnated with alcohol monoterpenes (PD2) was calculated as 35.9 min, followed by mixture of alcohol monoterpenes and citronella oil together (PD5) at 37.3 min. From these results the mixture of ketone monoterpenes showed the highest larvicidal effect, followed by alkene monoterpenes and then alcohol monoterpenes. The result showed that the knockdown time decreased with increased concentration in citronella oil. Mixing the citronella oil with any previous group decreased its adulticidal activity. The citronella oil alone showed medium effect. This result is in agreement with results obtained previously by Ramar and Paulraj who studied mosquito knockdown and adulticidal activities of essential oils by vaporizer, impregnated filter paper, and aerosol methods [29]. They proved that the citronella oil has adulticidal effect against* Cx. quinquefasciatus* and recorded KT_50_ of 11.4 min as determined by filter paper assay. In addition, Kawada et al. (2004) evaluated multilayer paper strip impregnated with metofluthrin against mosquitoes. They found that the use of metofluthrin reduced mosquito collection by > 80% during the 1st 4 weeks [24]. Fumigant bioassay of carvacrol, thymol, and l-perillaldehyde was conducted against* Cx. pipiens* [30]. They reported that carvacrol exhibited the highest fumigant activity followed by thymol and l-perillaldehyde, with LC_50_ values of 0.26, 0.28, and 0.34 mg/L air, respectively. In addition, Choi et al. determined the repellent activities of five monoterpenes to* Cx. pipiens*. They reported that terpinene had a potent repellent activity with a protection rate of 97% at a concentration of 0.05% topical treatment. Additionally, carvacrol and thymol showed an equivalent level of repellency. A spray-type solution containing 2% a-terpinene was tested for its repellent activity against* Cx. pipiens*. This solution showed stronger repellent activity than the currently used repellent, N,N-diethyl-z-methyl benzamide [31].

### 3.3. Fumigant Bioassay of Monoterpenes Incorporated on Wax

The results of the fumigant bioassay revealed that W1 showed the high knockdown effect (KT_50_ = 31.79) followed, in the descending order, by W2 (KT_50_ = 43.39) and then W7 (KT_50_ = 85.45). However, formulations of W3, W4, W5, and W6 showed neglected knockdown effect (KT_50_ > 120 min) (Table 3). These results proved that the ketone monoterpenes incorporated into wax gave the highest activity against mosquitoes. It can be noted that citronella oil decreased the activity of monoterpenes. This is due to the high density (*d* = 1.45 g/cm^3^) and less vapor pressure (VP) (VP = 0.15 mm Hg at 25°C) compared to the tested compounds (camphor, *d* = 0.992 g/cm^3^; VP = 4 mm, camphene, *d* = 0.842 g/cm^3^; VP = 2.4 mm, carvone, *d* = 0.96 g/cm^3^; VP = 0.4, fenchone, *d* = 0.948 g/cm^3^; VP = 0.16, menthone *d* = 0.895; VP = 0.5, geraniol,* d* = 0.889; VP = 0.2, limonene, *d* = 0.8411; VP = 5, menthol, *d* = 0.890; VP = 0.8, and thymol, *d* = 0.96; VP = 0.04).

Revay and his coauthors evaluated the tabletop mosquito repellent with another six commercially spatial repellents or mosquito traps under field conditions. They proved that, under minimal air-movement, three spatial repellent based products (ThermaCELL® Patio Lantern, OFF® PowerPad lamp, and Terminix® AllClear tabletop mosquito repellent) significantly reduced the biting-pressure (*t*-test, *P* < 0.01) when positioned at short distances from a volunteer (3, 7.5, and 10 ft.) [32].

### 3.4. Residual Activity of Monoterpenes against Female Adult of* Cx. pipiens*

Three single monoterpenes from different groups (camphor, menthol, and camphene) were evaluated against the adults of* Cx. pipiens* as determined by residual assay at different concentrations (5, 10, 20, and 40 mg/cm^2^) for 1 h exposure period. The results proved that, at 5 mg/cm^2^, all tested monoterpenes showed knockdown effect after more than one hour (Table 4). Among the three monoterpenes, menthol was the highest compound which gave KT_50_ of 11.66, 3.73, and 2.57 min at 10, 20, and 40 mg/cm^2^, respectively. However, camphor and camphene showed significant knockdown effect at the highest concentration (40 mg/cm^2^) with KT_50_ of 25.49 and 49.84 min, respectively. Deltamethrin was tested as a reference mosquitocide (Table 4). It showed 19.48, 12.02, 5.54, and 1.94 min at tested concentrations of 0.05, 0.1, 0.5, and 5 mg/cm^2^, respectively. These results are in agreement with the results obtained previously by Zahran and Abdelgaleil [5] who studied insecticidal and developmental inhibitory properties of monoterpenes on* Cx. pipiens*. They reported that menthol has more adulticidal activity than camphor and camphene with mortality percentages of 63.3, 53.3, and 10%, respectively, at 24 h after exposure time. The evaluation demonstrated that the efficacy of the monoterpenes (5, 10, 20, and 40 mg/cm^2^) was comparable with that of deltamethrin (0.05, 0.1, 0.5, and 5 mg/cm^2^). It can be noted that deltamethrin is still the most active against adult compared to the tested monoterpenes. However, this product (synthetic pyrethroid) does not meet the environment requirement and safety. Although a hundred times higher dosage than deltamethrin is required for monoterpenes to achieve the same effect as deltamethrin, the results give an insight into the potential use of monoterpenes compounds as safe and effective household insecticides for mosquito control, for the insecticides derived from plant sources have a much lower level of risk to the environment than synthetic pesticides [33]. However, further study for selecting more effective monoterpenes and optimizing the formulations should be conducted to enhance the efficacy and extend the effective duration. These results are in agreement with [30] who reported that the efficacy of the binary mixture of carvacrol and thymol (300 mg/mat) was comparable with that of* d*-allethrin (30 mg/mat).

## 4. Conclusion

The current study proved that the use of paper discs was better than the wax to formulate the monoterpene and produce ecofriendly formulations in the controlling of* Cx. pipiens*. Single or mixed monoterpenes showed high activity against* Cx. pipiens* adult. Activity of monoterpenes differed depending on the method of application. The selection of natural products reduces the environmental harmful impacts of pesticides and the phenomenon of resistance in adult mosquitoes. Finally such products can be used as ecofriendly alternative to chemical insecticides in vector-borne diseases control program.

## Figures and Tables

**Table 1:** Properties of paper discs impregnated in mixtures of monoterpenes.

Formulation code	Weight of disc before loading (mg) ± SD	Weight of disc after loading (mg) ± SD	Weight of pure loaded monoterpenes (mg) ± SD	% of monoterpenes loaded on paper discs formulations ± SD
PD1	200.41^a^ ± 7.60	214.27^a^ ± 11.24	13.86^a^ ± 5.61	6.380^bc^ ± 2.320
PD2	143.33^c^ ± 7.46	153.00^d^ ± 07.19	09.66^b^ ± 2.38	6.322^bc^ ± 1.571
PD3	152.47^c^ ± 5.36	161.20^c^ ± 09.61	08.72^c^ ± 5.66	5.264^c^ ± 3.096
PD4	145.93^c^ ± 7.71	160.33^cd^ ± 09.20	14.39^a^ ± 2.97	8.940^a^ ± 1.540
PD5	146.40^c^ ± 8.44	158.86^cd^ ± 09.13	12.46^ab^ ± 1.41	7.840^ab^ ± 0.743
PD6	166.53^b^ ± 21.42	178.33^b^ ± 23.94	11.79^ab^ ± 3.05	6.549^bc^ ± 1.052
PD7	170.33^b^ ± 17.73	179.53^b^ ± 18.45	09.19^b^ ± 1.15	5.132^c^ ± 0.496

Values are average of 15 replicates.

Weight of pure loaded monoterpenes (mg) = weight after loading (mg) − weight before loading (mg).

%  of  monoterpenes  loaded  on  paper  discs  formulations = (Weight  of  pure  loaded  monoterpenes  (mg)/Weight  of  disc  after  loaded  (mg)) × 100.

PD1: immobilization of blend of ketone monoterpenes (camphor, menthone, carvone, and fenchone) on paper discs.

PD2: immobilization of blend of alcohol monoterpenes (geraniol, thymol, and menthol) on paper discs.

PD3: immobilization of blend of alkene monoterpenes (limonene and camphene) on paper discs.

PD4: PD1 + citronella oil on paper discs.

PD5: PD2 + citronella oil on paper discs.

PD6: PD3 + citronella oil on paper discs.

PD7: immobilization of citronella oil on paper discs.

**Table 2:** Knockdown time (KT_50_) of different monoterpenes loaded on paper discs against female adult *Cx. pipiens*.

Compound	KT_50_^a^ (min)	95% confidence limits (min)		Slope^b^ ± SE	Intercept^c^ ± SE	(*χ* ^2^)^d^
		Lower	Upper			
PD1	17.200	14.800	19.400	5.786 ± 0.334	−7.152 ± 0.447	46.12
PD2	35.900	34.300	37.400	7.635 ± 0.421	−11.871 ± 0.667	15.281
PD3	27.445	25.919	28.913	8.419 ± 0.470	−12.111 ± 0.694	20.666
PD4	20.799	19.224	22.287	7.35 ± 0.433	−9.688 ± 0.595	24.426
PD5	37.329	36.035	38.639	6.286 ± 0.356	−9.882 ± 0.566	7.304
PD6	33.205	31.399	34.991	6.054 ± 0.329	−9.210 ± 0.514	16.426
PD7	28.726	26.764	30.651	4.897 ± 0.260	−7.141 ± 0.396	17.673

^a^Knockdown times required to kill 50% of the population exposed.

^b^Slope of the regression line ± standard error (SE).

^c^Intercept of the regression line ± SE.

^d^Chi square value.

PD: paper discs.

PD1: immobilization of blend of ketone monoterpenes (camphor, menthone, carvone, and fenchone) on paper discs.

PD2: immobilization of blend of alcohol monoterpenes (geraniol, thymol, and menthol) on paper discs.

PD3: immobilization of blend of alkene monoterpenes (limonene and camphene) on paper discs.

PD4: PD1 + citronella oil on paper discs.

PD5: PD2 + citronella oil on paper discs.

PD6: PD3 + citronella oil on paper discs.

PD7: immobilization of citronella oil on paper discs.

**Table 3:** Knockdown time (KT_50_) of different monoterpenes incorporated in wax against female adult *Cx. pipiens*.

Compound	KT_50_^a^(min)	95% confidence limits (min)		Slope^b^ ± SE	Intercept^c^ ± SE	(*χ* ^2^)^d^
		Lower	Upper			
W1	31.79	28.225	36.099	1.421 ± 0.129	−2.134 ± 0.191	4.741
W2	43.391	40.135	47.429	2.481 ± 0.183	−4.063 ± 0.282	10.148
W3	>120	—	—	—	—	—
W4	>120	—	—	—	—	—
W5	>120	—	—	—	—	—
W6	>120	—	—	—	—	—
W7	85.446	71.269	111.004	1.863 ± 0.197	−3.599 ± 0.306	5.256

^a^Knockdown times required to kill 50% of the population exposed.

^b^Slope of the regression line ± standard error (SE).

^c^Intercept of the regression line ± SE.

^d^Chi square value.

W: wax.

**Table 4:** Knockdown time (KT_50_) of single monoterpenes at different concentrations (mg/cm^2^) against the female adult *Cx. pipiens *as determined by residual assay.

Compound	Concentration (mg/cm^2^)	KT_50_	95% confidence limits (min)		Slop ± SE	Intercept ± SE	(*χ* ^2^)
			Lower	Upper			
Camphor	5	>60	—	—	—	—	—
	10	>60	—	—	—	—	—
	20	>60	—	—	—	—	—
	40	25.49	22.65	28.43	3.54 ± 0.198	−4.97 ± 0.296	28.67

Menthol	5	>60	—	—	—	—	—
	10	11.66	8.59	14.55	4.05 ± 0.22	−4.32 ± 0.27	87.40
	20	3.73	2.01	5.29	2.41 ± 0.23	−1.38 ± 0.25	23.43
	40	2.57	1.38	3.46	3.37 ± 0.66	−1.38 ± 0.55	1.79

Camphene	5	>60	—	—	—	—	—
	10	>60	—	—	—	—	—
	20	>60	—	—	—	—	—
	40	49.84	46.42	54.24	3.09 ± 0.23	−5.25 ± 0.37	9.76

Deltamethrin	0.05	19.48	16.86	21.87	4.42 ± 0.23	−5.70 ± 0.33	36.89
	0.1	12.02	10.11	13.81	3.78 ± 0.20	−4.08 ± 0.26	30.58
	0.5	5.54	5.01	6.03	5.53 ± 0.59	−4.11 ± 0.50	1.74
	5	1.94	0.59	2.98	3.08 ± 0.78	−0.89 ± 0.64	1.98
